# Methane emission from high latitude lakes: methane-centric lake classification and satellite-driven annual cycle of emissions

**DOI:** 10.1038/s41598-020-68246-1

**Published:** 2020-07-27

**Authors:** E. Matthews, Matthew S. Johnson, V. Genovese, J. Du, D. Bastviken

**Affiliations:** 1 Bay Area Environmental Research Institute @ NASA Ames Research Center, Moffett Field, Mountainview, CA 94035 USA; 2 NASA Ames Research Center, Moffett Field, Mountainview, CA 94035 USA; 3 California State University-Monterey Bay @ NASA Ames Research Center, Moffett Field, Mountainview, CA 94035 USA; 4 Numerical Terradynamic Simulation Group, University of Montana, Missoula, MT 59812 USA; 5 Department of Thematic Studies - Environmental Change, Linköping University, 581 83 Linköping, Sweden

**Keywords:** Carbon cycle, Limnology, Cryospheric science

## Abstract

Methane (CH_4_) is emitted from lakes by several processes: bubbles released from bottom sediments that reach the atmosphere (ebullition); spring release of CH_4_ trapped in bubbles in and under the ice during fall freeze (bubble release), and diffusion of CH_4_ from sediments to the surface. Each of these emission routes is highly variable over space and time, and episodic in the extreme, making reliable measurements difficult to carry out. However, lakes are receiving increasing interest for their important contribution to global CH_4_ emissions. Their area, distribution and emissions respond to interannual and longer-term climate fluctuations and close to half the world’s lake area is in high northern latitudes that are experiencing rapidly-warming temperatures and lengthening thaw periods. We report on a new spatially-explicit data set of lakes > 50°N, classified with methane-relevant criteria. The seasonality of daily CH_4_ fluxes is driven with satellite observations of thaw timing and duration. We found that observed thaw seasons are 10–30% shorter than those assumed in previous studies. The area of lakes is 1,095 × 10^3^ km^2^ and total CH_4_ emission is 13.8–17.7 Tg CH_4_ year^−1^: 11.2–14.4 Tg via diffusion and ebullition and 2.6–3.3 Tg from spring release of CH_4_ stored in bubbles in winter lake ice. This novel suite of data and methodologies provides a unique framework to model CH_4_ emission from lakes under current, past and future climates.

## Introduction

Lakes are increasingly recognized as potentially important contributors to methane (CH_4_) emissions. Similar to wetlands, their area, distribution and emissions are sensitive to interannual and longer-term climate fluctuations. About 40% of the world's lake area is in northern latitudes (> 50°N) that are experiencing rapidly-warming temperatures and longer thaw periods^1,2^.

Current estimates of CH_4_ emission from high-latitude lakes vary by a factor of two, from 12 to 25 Tg CH_4_ year^−1^ (Table 1)^3,7,8,9,10^. Only two studies are spatially- and temporally-explicit^9,10^ and simulate both emissions and thaw seasons (although only for the small area of thaw lakes north of 60°N). Other studies extrapolate mean fluxes derived from measurement compilations to estimates of lake areas^5,6,7,8^ or to one of two lake datasets^11,12^, and assume lengths of emitting thaw seasons to arrive at annual totals. Differences in methods defining lake areas, processes (diffusion, ebullition, release from bubble storage) and domains mean that results of these studies are not directly comparable. However, together they highlight fundamental gaps and uncertainties in estimates of CH_4_ emissions from lakes including the need for (1) spatially-explicit source characterization and spatial–temporal flux estimates; (2) estimates anchored in the large body of flux observations; (3) reduced uncertainties in timing and duration of thaw/flux seasons; and (4) inclusion of all relevant emission processes in emission estimates.

**Table 1:** Examples of studies of methane emission from lakes.

References	Description	Domain	Area, 10^3^ km^2^	Source of area	Processes*	Tg CH_4_ year^−1^
Holgerson and Raymond^4^	Flux and concentration obs. 427 lakes; modeled very small lake abundance; emissions modeled from concentration	Global	5,822	Verpoorter^12^ + modeled micro-lakes < 0.001 km^2^	D	16
Bastviken et al.^5^	Flux obs. 76 lakes, mean fluxes modeled; ebullition 365 days year^−1^, diffusion 224 days year^−1^	Global	2,803	Kalff^35^	D, Eb, B	8–48
Bastviken et al*.*^6^	Flux obs. 474 freshwater systems; extrapolate flux by lake type	Global	3,756	Downing^14^	D, Eb, B	88
Walter et al.^7^	Flux obs. 16 high-lat. lakes; assumed 120-day thaw season; doubled lake area to account for small lakes	> 45°N	1,055	GLWD^11^ small lakes < 50 km^2^ × 2	Ep	25.3 ± 10.7: D: 1.1 ± 0.2 Ep: 24.2 ± 10.5
Wik et al*.*^3^	Flux obs. 733 high-lat. lakes; classified lake types; applied mean daily fluxes and assumed thaw-season length to lake types	> 50°N	1,840	Verpoorter^12^ lakes < 5,000 km^2^	D, Eb: D × 2.2, B: (D + E) × 0.23	16.5 ± 9.1
Tan and Zhuang^9^	Thermokarst lakes are subset of prescribed lake distribution; lake biogeochemical model; spatially-temporally explicit fluxes	> 60 N	622	Kourzeneva^36^	D, Eb, Eh, B	11.86
Tan and Zhuang^10^	Coupled biogeochemical lake and thermokarst lake-evolution models; modeled thermokarst lake distribution; spatially- and temporally-explicit fluxes	> 60 N	259	Modeled	D, Eb, Eh, B	11.3
This study	CH_4_-centric lake classification; mean daily fluxes for lake types; satellite-derived thaw/emission season; spatially- and temporally-explicit fluxes	> 50°N	1,095	HydroLAKES + CCI-IW lakes < 5,000 km^2^	D, Eb, B	13.8–17.7 D + Eb: 11.2–14.4 B: 2.5–3.3

*D, diffusion; E, ebullition: b, background, p, point source, h, hotspot; B, bubble storage release.

This study addresses several of the gaps and uncertainties identified in existing estimates of CH_4_ emission from lakes. Specifically, we (1) developed a unique, spatially-explicit data set of lakes > 50°N classified into methane-relevant types consistent with a comprehensive suite of fluxes measured at boreal and Arctic lakes; (2) derived spatially-explicit thaw and freeze dates, and thus thaw seasons during which emissions occur, from daily satellite data of lake-ice phenology and landscape freeze–thaw dynamics and (3) estimated a full annual cycle of daily CH_4_ emissions for all lakes > 50°N. The main focus of the study is on diffusive and background ebullitive fluxes, but we also report on a simple estimate of the magnitude and timing of spring emissions of CH_4_ stored in bubbles in and under winter ice.

The following introductory sections analyze studies to date, with particular attention to causes for differences, and to potential over- and under-estimates inherent in the data and methodologies employed.

### Lake data and methane studies

Several lake data sets describing distribution of lakes by size^11,12^ have been used in CH_4_ studies (Table 1). The Minimum lake size in these data vary by several orders of magnitude: Holgerson and Raymond^4^ modeled abundance of lakes < 0.001 km^2^; Verpoorter et al.’s^12^ Landsat-based data set reports lakes down to 0.002 km^2^; whereas 0.1 km^2^ is the minimum lake size in the Global Lakes and Wetlands Dataset (GLWD)^11^ and in HydroLAKES^13^. These differences have important implications for CH_4_ studies due to abundance of small lakes^14^ and their recognized high CH_4_ fluxes due to their generally shallow structure that promotes CH_4_ bubbling from bottom sediments to reach the surface and be released into the atmosphere. In contrast, the majority of bubbles in larger lakes may be dissolved in the water column before reaching the surface^5,15^.

Areas are generally similar among data sets for lakes > 0.1 km^2^^11,12,13,14^ but areas for lakes from the satellite-derived data of Verpoorter et al.^12^ (hereafter Verpoorter) are 50–100% greater than in other datasets for all but the largest lakes meaning that studies relying on these data are likely to over-estimate emissions. The benefit of high-resolution satellite data^12,16,17^ to detect small lakes comes with the liability of capturing numerous unidentified non-lake features that artificially boost both ‘lake’ abundance and area.

### Methane emission from lakes

Two global and several high-latitude estimates of CH_4_ emission from lakes have been published. Table 1 summarizes examples of these studies, noting characteristics identified as possible contributors to differences among the emission results and to potential over- and under-estimates emanating from the data and methods employed.

High-latitude emissions range from 12 to 25 Tg CH_4_ year^−1^^3,7,8,9,10^ (Table 1) and only two of these studies are spatially-temporally explicit^9,10^. Studies typically apply fluxes averaged from measurements to estimates of lake areas^3,4,7,8^ and assume lengths of emitting thaw seasons. Bastviken et al.^5^ modeled fluxes from ~ 75 flux and ancillary measurements for estimates of lake area, assuming a 365-day ebullition season and 224 days for diffusion during an average thaw season. This first global estimate was 8–48 Tg. The study includes all major emission processes but the global lake area is lower than others which likely depresses total emission. Bastviken et al.^6^ relied on more measurements and larger lake areas^14^ resulting in a global total which increased markedly (8–48 to 88 Tg) with more comprehensive data. Holgerson and Raymond^16^ modeled emissions from simultaneous measurements of flux and concentration applied to Verpoorter areas augmented with model estimates of microlakes < 0.001 km^2^ (Table 1). Global CH_4_ emission via diffusion is 16 Tg year^−1^ with very small lakes accounting for just ~ 9% of area but ~ 40% of emission. This estimate comprises only diffusive fluxes that may account for only 10–50% of total emissions^3,18^ suggesting that global emissions comprising all processes (Table 1) may be 3–5 times this value. At the same time, 16 Tg may overestimate diffusive emissions due to high lake areas of Verpoorter.

Wik et al.^3^ (hereafter Wik) also relied on the large Verpoorter areas but excluded lakes > 5,000 km^2^ assuming these larger lakes, which are also deeper, produce little or no CH_4_. They defined lake types by environmental and geophysical characteristics including formation process, permafrost state and underlying sediments. Wik then applied mean daily diffusive (D) thaw-season fluxes (Table 1) and estimated thaw-season lengths for lake types; background ebullitive (Eb) fluxes were uniformly included as (D × 2.2) making Eb ~ 55% of annual (D + E) emissions, and release of CH_4_ stored in bubbles (B) in and under lake ice was assumed to add 23% to thaw-season (D + E) emissions. The flux from lakes > 60°N totaled to 16.5 Tg CH_4_ year^−1^. Although Wik relied on large Verpoorter areas for most lake types, this potential overestimate is moderated by limiting emitting area to lakes < 5,000 km^2^. However, thaw seasons are ~ 10–30% longer than those observed for the same lake types in this study suggesting a high bias. Lastly, all emission processes except for highly uncertain hot-spot ebullition are included in the estimate, albeit in simple ways.

Other studies focusing on high-latitude thaw lakes^7,8^ estimate thermokarst fluxes, comprising additional ebullition-related processes reporting fluxes as high as ~ 24 Tg CH_4_ year^−1^ north of 45°N primarily via point-source and hotspot ebullition with only 1 Tg via diffusion (Table 1). These values for a subset of high-latitude lakes approach the total emission inferred for all high-latitude biogenic sources (natural wetlands and lakes) from model inversions^19^ suggesting a possible inconsistency with atmospheric measurements of CH_4_ concentrations.

Tan and Zhuang^9^ applied a biogeochemical lake-methane model to thermokarst lakes > 60°N; lake area and distribution were prescribed; a follow-up study^10^ coupled the lake model with a thermokarst-lake evolution model (Table 1). These studies report high-latitude thermokarst emissions of 11.86 and 11.3 Tg CH_4_ year^−1^ but neither accounts for very small (< 0.1 km^2^) or non-thermokarst lakes; thermokarst lakes may account for only 15–30% of high-latitude lake area (Wik; this study). We surmise that adding emissions for the remaining 70+ % of lakes and all wetlands may exceed top-down constraints on total high-latitude biogenic emissions^19^.

Analysis of this sample of global and high-latitude studies highlights fundamental differences among lake-methane studies with respect to domain, lake area and distribution, emission processes and lake types. While some of these differences are attributable to known factors, and can be improved upon, the lake source remains very uncertain regarding flux quantification.

## Data and processing

A range of native spatial resolutions characterizes the data sets used in this study. Each was gridded at the reference resolution of 0.25° latitude × 0.25° longitude.

### Daily methane fluxes

Wik described high-latitude lake types, i.e., glacial, thermokarst, peat pond, beaver ponds, and assigned mean daily CH_4_ flux rates to these types derived from 733 flux observations. We adopted Wik’s mean daily fluxes for lake types in common between these studies; our new organic lake type was assigned the peat-pond flux while the new ‘other’ class is assigned a flux similar to glacial lakes; due to the uncertainty regarding characteristics of these ‘other’ lakes, we also estimated emissions by doubling their daily flux, from 0.05 to 0.1 g m^−2^ day^−1^, since the initial estimate employed a very conservative value for these substantial areas. Daily CH_4_ fluxes range from 0.045 to 0.145 g m^−2^ days^−1^ (Table 2).

**Table 2:** Area, classification criteria, thaw-season length and methane emission (diffusion, background ebullition and bubble storage) for lakes < 5,000 km^2^ north of 50°N.

Lake type	Area, 10^3^ km^2^	SOC* kg C m^−2^	Permafrost state^#^	Ground ice % vol	Mean thaw season, days	Daily flux, g m^−2^	Annual flux, g m^−2^	Annual emission, Tg year^−1^
1. Thermokarst	224	All	C, D	≥ 10	108	0.121	13.2	3.1
2. Glacial	352	All	C, D	< 10	115	0.045	5.2	1.9
			S, I	≥ 10				
3. Peat pond	69	≥ 10	S, I	< 10	166	0.145	24.3	1.7
4. Other organic	48	≥ 10			180	0.145	26.2	1.3
5. Other	402	All			155	0.05–0.1	6.8–13.6	3.2–6.4
Subtotal D + Eb								11.2–14.4
Bubble flux								2.6–3.3
Total	1,095							13.8–17.7

*Depth-weighted 0–100 cm soil.

^#^ C (continuous), D (discontinuous), S (sporadic), I (isolated).

### Methane-emission seasonality

We relied on two satellite-based microwave data sets to calculate thaw and freeze dates, and thus the timing and length of the thaw season, to drive methane-emission seasonality. Daily flux rates for lake types (Table 2) were applied to relevant lake areas for the duration of the thaw season such that the annual emission cycle is determined by the seasonality of emitting lake areas. We employed climatological thaw and freeze dates, i.e., mean thaw and freeze dates calculated for all years of each data set and averaged to a climatology that reflects typical conditions and maximizes data available to define lake-ice phenology and freeze–thaw dynamics. Emissions commence on local thaw dates and end on local freeze dates; the difference between them defines thaw-season length, and the duration and timing of the emission season.

#### Lake ice phenology (LIP)

The lake-ice phenology data^20^ (2002–2015) were developed from the Advanced Microwave Scanning Radiometer - Earth Observing System (AMSR-E) microwave instrument aboard the Moderate Resolution Imaging Spectroradiometer (MODIS) Aqua satellite (2002–2011) and the Advanced Microwave Scanning Radiometer 2 (AMSR2) instrument on the Japan Aerospace Exploration Agency (JAXA) Global Change Observation Mission 1-Water (GCOM-W1) satellite for the latter years. The data comprise a subset of Northern Hemisphere lakes > 50 km^2^_;_ the remainder were excluded by the authors due to bad or missing data.

#### Freeze–thaw dynamics (FT)

We employed daily freeze–thaw dynamics from the satellite microwave data of Kim et al.^21^ (2003–2015) for all lakes not in the LIP data set; we used version 4 (FTv04) that encompasses a larger domain, and does not mask out lakes, as done in earlier versions. The data set was developed from multifrequency, dual polarization brightness-temperature measurements from the Special Sensor Microwave Imager (SSM/I) and Special Sensor Microwave Imager/Sounder (SSMI/S), passive microwave radiometers which are aboard Defense Meteorological Satellite Program (DMSP) satellites.

### Permafrost and ground ice

Distribution and type of permafrost and ground-ice content were derived from Brown et al.^22^ and used in the classification of lake types (Table 2). We note that an alternative global data set^23^ of 1-km permafrost distribution and type based on satellite observations and the TTOP (temperature at the top of permafrost) model was published after substantial work on this project was completed, thus we were not in a position to evaluate or use it although the general permafrost patterns are similar to Brown et al.^22^.

### Soil organic carbon (SOC)

We relied on the Harmonized World Soil Database (HWSD)^24^ to calculate SOC content per square meter, depth-weighted for the top 100 cm of soil, as a classification criterion for lake types (Table 2).

### Lake distribution and area

An important component underlying this study is a new global lake data set composed of HydroLAKES^13^ for lakes > 0.1 km^2^, augmented with ~ 6.5 million smaller lakes (0.02–0.1 km^2^) extracted from Lamarche et al.^16^ after excluding non-lake water bodies in order to isolate small lakes. Lamarche et al**.**^16^ is the European Space Agency’s Climate Change Initiative Inland-Water remote-sensing dataset (CCI-IW). River areas were removed from CCI-IW using the Global River Widths data derived from Landsat (GRWL)^25^; reservoirs were removed using Global Reservoirs and Dams (GRanD)^26^ that identifies ~ 6,800 larger reservoirs and the GOOD^2^ data set^27^ providing information for ~ 35,000 smaller reservoirs. The ~ 6.5 million remaining water bodies < 0.1 km^2^ were merged with HydroLAKES.

## Results and discussion

### Lake area and distribution

**Figure 1:**
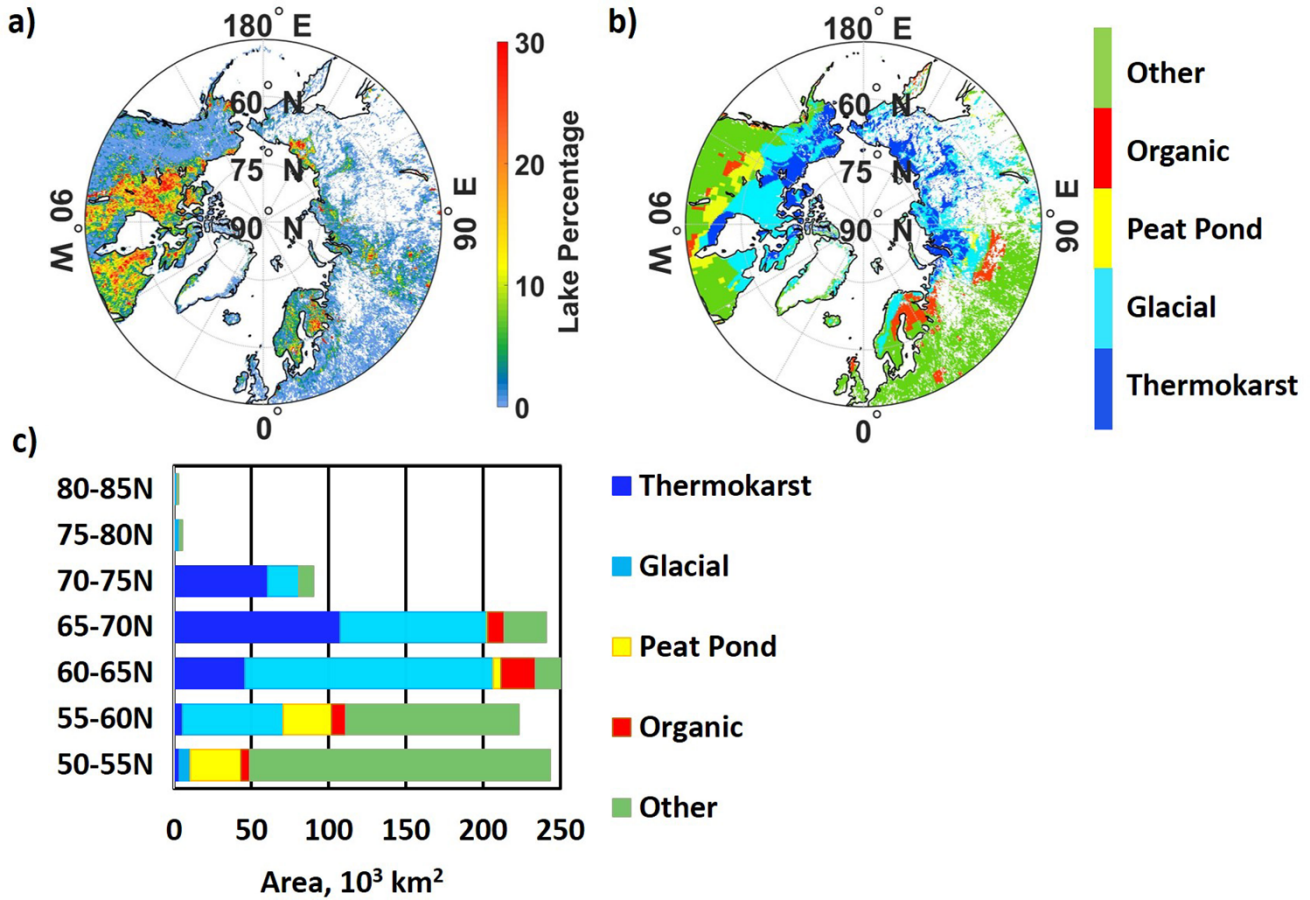
(**a**) Lake percentage, (**b**) classification of lake types, and (**c**) latitudinal areas by lake type. Total lake area is 1,095 × 10^3^ km^2^. White areas in Figs. (**a**) and (**b**) denote zero lake percentage. All maps are at 0.25°lat/lon resolution.

The total area of lakes < 5,000 km^2^ and north of 50°N is 1,095 × 10^3^ km^2^ (Table 2, Fig. 1a). Dense swaths of lakes occupy eastern and central North America where they are interwoven with natural wetlands. Numerous lakes covering small fractions of cells are found in western North America, western and central Europe, along the northern coast of Russia and in the Siberian Lowlands. These landscapes are also occupied by small wetlands that are often interwoven with lakes which has caused difficulties in distinguishing between them. A large majority of microlakes from CCI-IW occur north of 50°N.

### Methane-centric classification of lakes

The lake dataset is the basis for classifying lakes with methane-centric criteria (Sect. 3 and Table 2), focusing on the region > 50°N because the majority of flux measurements were obtained in this area and Wik provide excellent guidance for classifying methane-relevant lakes in boreal and Arctic environments.

We generally followed the scheme of Wik to classify lakes but introduced the following modifications: (1) Wik used only areas, not the spatial distribution, of Verpoorter and assumed fractions of high-latitude lake area to be thermokarst lakes, glacial-postglacial lakes and peat ponds based on areal estimates from other studies. In contrast, we directly classified the spatial distribution of lakes at 0.25° lat/lon resolution; (2) our domain (> 50°N) extends farther south than that of Wik and encompasses some lake characteristics not addressed in their classification, i.e., lack of permafrost and ground ice. We therefore added ‘organic’ and ‘other’ lakes to the classification (Table 2).

We employed the spatial distribution of criteria variables to implement the classification. Since all lake and criteria data are at the same 0.25° lat/lon resolution, each grid cell is classified as a single lake type. The result of lake classification, and criteria and thresholds for data used to classify them, are shown in Table 2; the distribution of lake types and their latitudinal areas are shown in Fig. 1b, c. As expected, thermokarst and glacial lakes dominate north of 60°N, while organic and other lakes dominate 50°–60°N. Lake areas are about equally distributed between 50°N and 70°N, with a maximum at 60–65° and a sharp decline north of 70°N.

### Satellite-derived timing and duration of thaw (and emission) season

**Figure 2:**
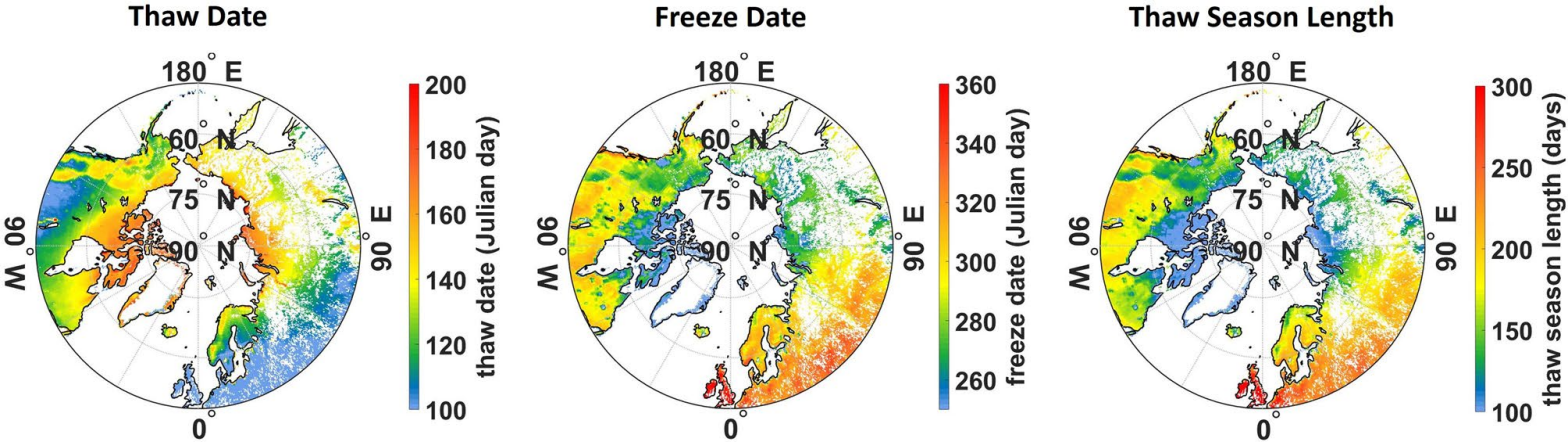
Seasonality and duration of emissions for lake locations: thaw date (left), freeze date (middle), length of thaw season (right), i.e., number of days between thaw and freeze dates. Note difference in scales for each panel. All maps are at 0.25° lat/lon resolution.

Lake-methane studies that are not spatially explicit ^3,4,5,6,7,8^ must estimate thaw-season lengths from field observations. This study employed daily satellite observations of lake-ice phenology^20^ and freeze–thaw dynamics^21^ to drive the seasonality of daily lake fluxes by lake type. Mean thaw-season lengths for types are shown in Table 2; actual thaw seasons depend on local thaw and freeze dates. Distributions of satellite-derived thaw and freeze dates, and thaw-season length, are depicted in Fig. 2a–c. Mean thaw seasons from this study are shorter by 10–30% than Wik’s for the same lake types. Wik’s assumed thaw seasons are 149, 171 and 185 days for thermokarst, glacial and peat lakes, respectively, while satellite-derived season lengths for the same lakes from this study are 108, 115 and 166 days (Table 2). These differences highlight the uncertainty in the length of thaw seasons that exert very strong influences on emissions. Wik’s thaw seasons may be more similar to the lengthened thaws anticipated under future warming, further suggesting a potential emission overestimate from this study. The differences are partly explained by Wik’s application of a single thaw-season length for each lake type whereas Fig. 1b shows that thaw seasons are latitudinally dependant, e.g., glacial lakes extend over 20° of latitude and observations reveal that season length declines by ~ 30 days with a 10° increase in latitude (Fig. 2c). These results confirm that annual emissions are sensitive not only to lake area and daily fluxes but also to lake distribution which determines the length of thaw and emission seasons. Spatially-explicit data sets are needed to realistically capture this important variable in methane studies.

### Spatial and temporal methane emissions

**Figure 3:**
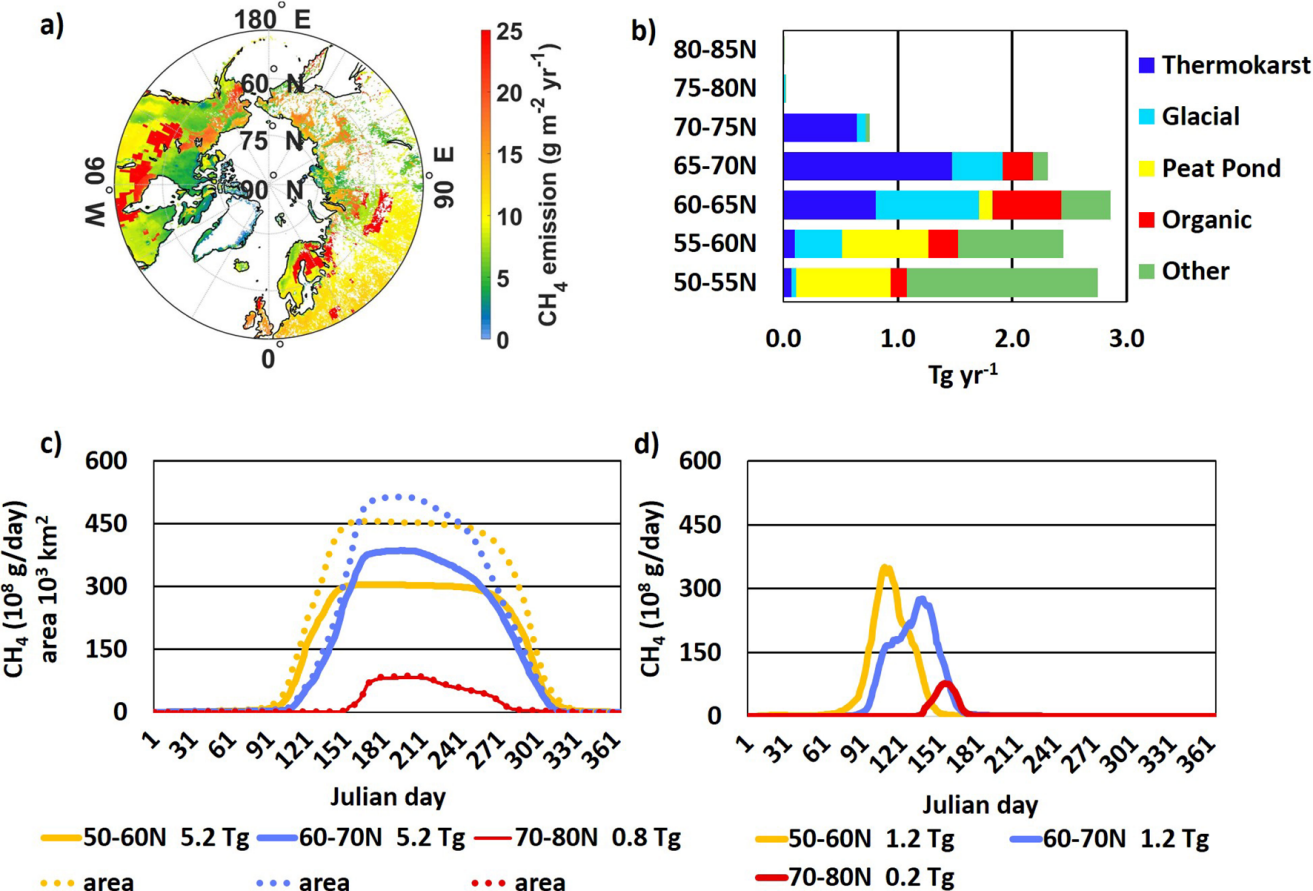
Annual methane emission (D + Eb) from high-latitude lakes: (**a**) distribution of annual emissions (0.25° lat/lon), (**b**) latitudinal emission by lake type, (**c**) daily methane emission and emitting area by latitude zone and (**d**) daily bubble flux by latitude zone.

Annual CH_4_ emission from high-latitude lakes is the product of daily flux and length of the local thaw-season (Fig. 3c). Values range from ~ 4 g CH_4_ m^−2^ year^−1^ for high-Arctic glacial lakes characterized by low fluxes and short seasons to > 25 g CH_4_ m^−2^ year^−1^ for organic lakes at lower latitudes with extended seasons (Table 2). A comparison of the latitudinal distribution of areas (Fig. 1c) and emissions (Fig. 3b) illustrates the large impact of productive peat ponds that account for just 6% of area but 17% of emissions. Similarly, thermokarst lakes cover 20% of total lake area and emit 28% of methane despite their short thaw seasons.

The seasonal cycle of annual (D + Eb) lake fluxes (Fig. 3c) illustrates latitudinal differences in magnitude and seasonality of emissions even for 10° latitude zones. The flux season at 50–60°N begins in April and ends by the start of November with sustained emissions of 300 × 10^8^ g CH_4_ days^−1^ at high season. Emissions for 60°–70°N begin about a month later, maintaining a higher maximum daily flux of ~ 375 × 10^8^ g CH_4_ governed by the larger emitting lake area of ~ 525 × 10^3^ km^2^ from early June through early August followed by a gradual decline to zero by the beginning of November. Lakes north of 70°N emit little methane due to the combined influence of small area and a short thaw season starting in mid-June, rising to a maximum daily flux of ~ 75 × 10^8^ g CH_4_ for two months, and declining to zero by early October. Figure 3d shows the seasonality of the bubble flux by latitude zone. These fluxes (equal to 23% of annual (D + Eb)) are of short duration, i.e., we assumed they start 14 days before local thaw dates. These emissions are very uncertain and are currently supported by few measurements. However, they may play a role in the dynamics of late winter-early spring atmospheric CH_4_ concentrations.

### Monthly fluxes

**Figure 4:**
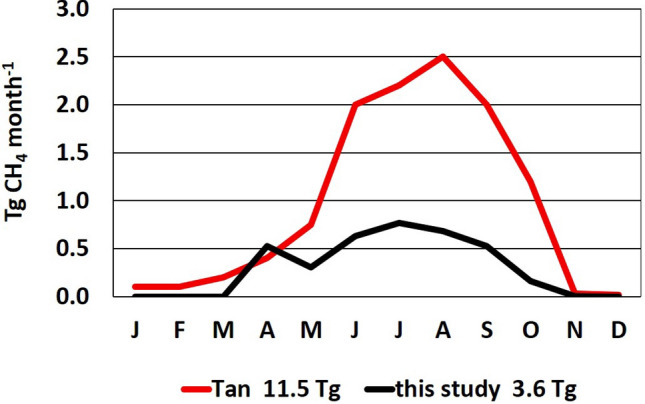
Monthly methane emission from thermokarst lakes > 60°N from Tan and Zhuang^10^ (red line, annual total = 11.5 Tg) and from this study (black line, annual total = 3.6 Tg).

The only study with which to compare our spatial–temporal lake emissions is that of Tan and Zhuang^10^ who modeled emissions from thermokarst lakes > 60°N, a subset of our lakes and domain. Figure 4 compares monthly emissions from thermokarst lakes > 60°N from Tan and Zhuang^10^ and from this study. Since bubble fluxes are included in the modeled emission^10^, we added a bubble flux to this study’s (D + Eb) flux from thermokarst lakes > 60°N as described above.

Areas of thermokarst-lake > 60°N are similar in the two studies (Tables 1, 2) but modeled thermokarst emissions^10^ are 11.5 Tg year^−1^, more than triple the 3.6 Tg from this study implying mean annual fluxes of 43 and 12 g CH_4_ m^−2^ year^−1^, respectively. This high modeled flux exceeds substantial fluxes for Siberian thaw lakes^7^ by 20%. The large difference in thermokarst emissions is due in part to different processes included in the studies. Both report D, Eb and B but Tan and Zhuang^10^ also model hotspot ebullition (Table 1). However, it’s unlikely that this process can explain the substantial difference shown in Fig. 4. Moreover, this high thermokarst emission may be inconsistent with high-latitude constraints on total biogenic CH_4_ emissions when emissions for other lakes and for all wetlands are factored in.

Timing of annual maxima also differ between the studies: maximum emission driven by satellite observations occurs in July for this study, and in August for modeled emissions^10^. The August maximum may be due to maximum methane-producing areas in that month and/or maximum fluxes per square meter of emitting areas. Nonetheless, a month is a substantial difference in high latitudes with short thaw seasons.

## Significance and conclusions

We estimated annual CH_4_ emission from lakes via diffusion and background ebullition to be 11.2–14.4 Tg and release of bubble storage to be 2.5–3.6 Tg, totaling 13.8–17.7 Tg (Tables 1, 2).

These results relied on a new lake data set developed for this study comprising HydroLAKES^13^ for lakes > 0.1 km^2^ augmented with ~ 6.5 million smaller lakes (0.02–0.1 km^2^) extracted from a high-resolution remote-sensing data set^16^. These combined data are the centerpiece of the first spatially-explicit, methane-centric classification of boreal and arctic lakes, demonstrating that existing classification concepts based on available observations can be implemented to classify lakes for CH_4_ studies and ultimately link the distribution of lake types with measured fluxes for those same types. We acknowledge that a substantial fraction of lakes, especially 50–60°N, remain ‘other’ (unclassified). Additional criteria are expected to improve the classification including, *inter alia*, lake size and depth, topography and yedoma extent.

This study represents the first use of satellite observations to introduce realistic thaw seasons to drive daily fluxes, thus reducing uncertainties inherent in assumptions of constant season lengths for widely-distributed lakes. We found that observed thaw seasons are shorter by ~ 10–30% (19–56 days among lake types) than those of Wik. In fact, the Wik may be more similar to the extended thaw periods anticipated under a warming climate, further suggesting that this variable may contribute to over-estimating emissions from high-latitude lakes. This finding highlights the crucial need to quantify baseline interannual variability of thaw seasons from decadal observations^20,21^ in order to improve the realism of emission estimates for individual years in the present and to identify trends and predict future emissions. The satellite observations may also improve methods to predict future or past thaw seasons from climate data.

We acknowledge it may be unrealistic to assume that lakes > 5,000 km^2^ do not emit any CH_4_ as done by Wik and in this study. CH_4_ emissions have been measured in Lake Ontario^28^ and Lake Erie^29,30^, and surface waters supersaturated with CH_4_ were observed in several areas of Lake Michigan^31^. To assess the potential impact of including shallow areas of large lakes as emitting surfaces, we assumed that 10% of the area of lakes > 5,000 km^2^ emit CH_4_ at the daily flux rates of their classified lake types for the duration of local thaw seasons. Annual emitting area and emission > 50°N rose only ~ 1.5%, specifically by 16 × 10^3^ km^2^ and 0.2 Tg year^−1^. These changes are small due to the scarcity of large lakes in high latitudes but including shallow regions of large lakes in lower latitudes will result in larger relative increases in area and emission.

The work of Tan and Zhuang^10^ modeling thermokarst lake initiation and evolution, together with emissions, represents an important approach to predict future emissions under warmer climate, namely transitions between lake types. The model is now limited to thermokarst lakes but expanding it to all lakes and possible transitions would represent a major step toward quantifying the current and future role of lakes in the global CH_4_ cycle especially because thaw lakes and their emissions are expected to continue increasing in the near term^32^, likely followed by drainage and decline^,^^33^. A more widespread climate impact on future lake emissions is longer thaw seasons that are already occurring^34^, since thaw season is acknowledged as one of the primary determinants of annual fluxes.

A comprehensive assessment of the role of high-latitude lakes in the global CH_4_ cycle requires more rigorous quantification of emissions from point and hotspot ebullition which may be achieved by additional, targeted field measurements and incorporation of spatially-explicit lake area and type, particularly for very small lakes that are more likely to be thermokarst features with high ebullitive emissions. Spatially-explicit datasets of lake types linked with flux measurements for these types makes it possible to exploit the full scope of the large body of field observations to improve emission estimates.

An important advantage of spatially- and temporally-explicit emissions is the opportunity to assess their realism via inverse-model studies^19^ by incorporating independent lake and wetland biogenic CH_4_ sources that are intertwined across landscapes and whose emissions are both sensitive to climate change.

Our new spatially-explicit data sets and methods provide a unique framework to study and model CH_4_ emissions from lakes in novel ways. Specifically, the methane-centric classification of lake types provides the capacity to model their transitions over time; incorporation of satellite observations to define timing and duration of thaw periods—and thus of flux seasons—make it possible to benchmark interannual variations in thaw seasonality, and detect trends over longer time periods. The classified lake data set and associated emissions presented here are mutually exclusive of complementary wetland and reservoir data and emissions which will be included independently in future versions of our data set. This suite of data and methodologies will make it possible to investigate the individual roles of wetland, lake and reservoir emissions in the global CH_4_ cycle which has not been possible to date.
